# CircZNF609 enhances hepatocellular carcinoma cell proliferation, metastasis, and stemness by activating the Hedgehog pathway through the regulation of miR-15a-5p/15b-5p and GLI2 expressions

**DOI:** 10.1038/s41419-020-2441-0

**Published:** 2020-05-12

**Authors:** Yangke He, Hui Huang, Li Jin, Fang Zhang, Ming Zeng, Liang Wei, Shijia Tang, Dongqin Chen, Wansheng Wang

**Affiliations:** 1 Cancer Center, Sichuan Academy of Medical Sciences and Sichuan Provincial People’s Hospital, 610072 Chengdu, Sichuan China; 2 Cancer Center, Sichuan Provincial People’s Hospital, University of Electronic Science and Technology of China, 611731 Chengdu, China; 3 Department of Radiotherapy, Sichuan Cancer Hospital & Institute, Sichuan Cancer Center, School of Medicine, University of Electronic Science and Technology of China, 55# Renmin South Road, 610041 Chengdu, Sichuan China; 4 Department of Burn and Plastic Surgery, Affiliated hospital of Chengdu Medical College, 610000 Chengdu, Sichuan China; 5 Department of Medical Oncology, Renji Hospital, School of Medicine, Shanghai Jiao Tong University, No. 160 Pujian Road, Pudong New District, 200127 Shanghai, China; 6 Department of Medical Oncology, The Affiliated Cancer Hospital of Nanjing Medical University&Jiangsu Cancer Hospital&Jiangsu Institute of Cancer Research, No. 42 Baiziting Road, Xuanwu District, 210009 Nanjing, China; 7 Department of Medical Oncology, the First Affiliated Hospital of Soochow University, No.188, Shizi Street, Gusu District, 215006 Suzhou, Jiangsu China; 8 Department of Interventional Radiology, the First Affiliated Hospital of Soochow University, No. 188, Shizi Street, Gusu District, 215006 Suzhou, Jiangsu China

**Keywords:** Cancer, Cell biology

## Abstract

Emerging evidence has revealed that aberrantly expressed circular RNAs (circRNAs) play vital roles in tumorigenesis and progression of diverse human malignancies. Although an existing literature has elucidated the regulatory role of circZNF609 in breast cancer, the crucial function that circZNF609 exerted on hepatocellular carcinoma (HCC) remains unclear. Herein, we determined to explore the molecular mechanism of circZNF609 in HCC. In this study, circZNF609 was conspicuously overexpressed and featured with loop structure in HCC. Functional tests revealed that decreased expression of circZNF609 suppressed cell proliferation, metastasis and stemness, whereas induced cell apoptosis in HCC. Subsequent molecular mechanism assays indicated that circZNF609 contributed to HCC progression through activation of Hedgehog pathway. Moreover, circZNF609 was found to be negatively correlated with miR-15a-5p/15b-5p but positively correlated with GLI2. Moreover, there was a negative correlation between miR-15a-5p/15b-5p and GLI2. Rescue experiments testified that GLI2 overexpression could recover circZNF609 depletion-mediated function on HCC development while miR-15a-5p/15b-5p inhibition could partially rescue circZNF609 silencing-mediated effect on HCC progression. Final experiments in vivo further elucidated the suppressive function of circZNF609 knockdown on the tumorigenesis of HCC. Briefly, circZNF609 enhances HCC cell proliferation, metastasis, and stemness by activating the Hedgehog pathway through the regulation of miR-15a-5p/15b-5p and GLI2 expressions.

## Introduction

Deemed as the most common primary hepatic malignancy, hepatocellular carcinoma (HCC) is a big threat to human health globally^1,2^. It is the sixth most prevalent cancer and the third major cause of cancer-associated mortality all around the world^2,3^. Yearly, about 750,000 new cases are determined and ~500,000 death cases are related to HCC^4^. Getting infected with hepatitis B or C virus, abuse of alcohol, cirrhosis, as well as ingestion of aflatoxin B1, have been identified as the common risk factors that induce hepatocarcinogenesis^5–9^. Although in the past years, surgical resection has been the major choice for sick person with respectable HCC, tumor recurrence rate is still high after surgery because of the refractory feature of tumor^10^. In spite of strategies adopted to restrain HCC metastasis, no obvious positive effects have appeared^11^. Thus, therapies to prevent the recurrence and metastasis of HCC are still urgently needed. Understanding molecular mechanisms underlying HCC and probing into new therapeutic targets for HCC are crucial at present.

Emerged as a novel type of RNA, circular RNA (circRNA) is characterized with a closed loop structure without 5′–3′ polarity and apt to be upregulated in the cytoplasm of eukaryotic cells^12,13^. CircRNA is largely featured with stability, abundance and conservation, and it is generally expressed in specific tissues or at a particular developmental time^14,15^. CircRNA is also considered to exist prevalently in mammals and is primarily associated with gene regulation in vivo^13,16,17^. A number of studies have uncovered the critical impact that circRNA elicits on the initiation and progression of different kinds of diseases, particularly in human malignancies. For example, hsa_circRNA_0006528 facilitates the proliferation, invasion, and migration of breast cancer cells through targeting miR-7-5p/Raf1 axis^18^. Circ_0001721 indicates poor prognosis in osteosarcoma and drives osteosarcoma progression by sponging miR-569 and miR-599^19^. Hsa_circ_0020123 elevates ZEB1/EZH2 expression to promote non-small cell lung cancer progression by competitively binding with miR-144 and inhibiting miR-144 expression^20^. Intriguingly, existing evidence has also manifested the regulatory role of circRNAs in HCC. For instance, hsa_circ_101280 accelerates HCC cell growth via regulation of miR-375/JAK2 axis^21^. The circRNA circZNF609 whose circBase ID is hsa_circ_0000615, is located at chr15: 64791491–64792365. According to a former study, circZNF609 elicits significant function on myoblast proliferation^22^. Besides, it has been revealed to sponge miR-150-5p to regulate AKT3 expression in Hirschsprung’s disease^23^. Strikingly, a previous investigation has clarified that circZNF609 facilitates cell growth and metastasis by sponging miR-145-5p to elevate p70S6K1 in breast cancer^24^. However, the specific role of circZNF609 in HCC has not been explored up to now. Herein, it’s worth detecting the performance of circZNF609 in HCC.

The purpose of this current study was to make exploration of the specific function that circZNF609 exerted on HCC development, along with its molecular mechanism. All the findings from this study conclude that circZNF609 enhances HCC cell proliferation, metastasis, and stemness by activating the Hedgehog pathway through the regulation of miR-15a-5p/15b-5p and GLI2 expressions, shedding new light on exploring efficient targets for HCC treatment.

## Materials and methods

### Tissue samples collection

From 2013 to 2018, HCC tissues and peri-tumor tissues of 49 patients who had not received local or systemic therapy prior to surgery were acquired from Sichuan Academy of Medical Sciences and Sichuan Provincial People’s Hospital. Samples were frozen in liquid nitrogen at once and stored at –80 °C. All patients signed the informed consent. All protocols were approved by the Ethics Committee of Sichuan Academy of Medical Sciences and Sichuan Provincial People’s Hospital.

### Cell culture

HCC cell lines (HepG2, Huh-7, HCCLM3, MHCC-97H) and normal liver epithelial cell line (THLE-3) were obtained from Shanghai Institute of Cell Biology (Shanghai, China) and maintained at 37 °C in 5% CO_2_. Cells were cultured in RPMI-1640 (Thermo Fisher Scientific, Waltham, MA, USA) containing 10% fetal bovine serum (FBS; Thermo Fisher Scientific), streptomycin (100 μg/ml) and penicillin (100 U/ml). SAG was obtained from Sigma-Aldrich (St. Louis, MO, USA).

### RNA extraction and quantitative reverse transcription PCR (RT-qPCR)

RT-qPCR analysis was carried out as described previously^25^. GAPDH/U6 was used as internal control.

### Cell transfection

HCCLM3 or MHCC-97H cells were plated in six-well plates for transfection with Lipofectamine2000 (Invitrogen, Carlsbad, CA, USA). Short hairpin RNAs (shRNAs) against circZNF609 (sh-circZNF609#1/2), miR-15a/15b-5p mimics, miR-15a/15b-5p inhibitor, along with sh-NC, NC mimics and NC inhibitor were constructed by Obio Technology (Shanghai, China). GLI2 overexpressing plasmid and empty pcDNA3.1 vector were also obtained from Obio Technology. After 48 h, cells were collected.

### Microarray and KEGG analyses

Differentially expressed genes (DEGs) were screened out after silencing circZNF609 by whole genome microarray expression profiling in line with the threshold of log2 (fold change) >2 and *P*-value < 0.05. KEGG analysis was performed to identify the related enriched pathways in response to circZNF609 depletion.

### Nucleic acid electrophoresis

The complementary DNA (cDNA) and genomic DNA (gDNA) PCR products of circZNF609 were determined using TE buffer AGAR gels (Thermo Fisher Scientific). DL600 (KeyGen, Nanjing, China) was set as DNA marker. The electrophoretic voltage (110 V) was adopted to separate the DNA half an hour, followed by UV irradiation.

### Actinomycin D and RNase R treatment assay

Actinomycin D (Merck-Millipore, Darmstadt, Germany) and RNase R (Epicenter Biotechnologies; Madison, WI, USA) were used for treating cells, followed by RT-qPCR analysis.

### Cell counting kit-8

Transfected cells in 96-well plates were cultured with CCK-8 solution (Dojindo, Tokyo, Japan) for indicated times, absorbance at 450 nm was determined using a microplate reader (Bio-Tek Instruments, Winooski, VT, USA).

### Colony formation assay

Transfected cells were plated into 6-well plates for two weeks. Colonies were fixed and stained. Colony numbers (≥50 cells) were counted.

### Sphere formation assay

Transfected HCCLM3 or MHCC-97H cells were added into 96-well plates, followed by cultivation. The spheroids were photographed via an optical microscope (Olympus, Tokyo, Japan).

### Flow cytometry analysis

Apoptosis rate was analyzed using a FACSCalibur™ fow cytometer (BD Biosciences) as per previously mentioned^26^.

### TUNEL assay

Apoptosis of HCCLM3 or MHCC-97H cells after transfection were assessed by TUNEL as mentioned before^27^.

### Western blot analysis

As previously described^28^, primary antibodies against Bcl-2 (ab32124), Bax (ab32503), cleaved caspase-3 (ab2302), total-caspase-3 (ab13847), cleaved caspase-6 (ab2326), total-caspase-6 (ab185645), cleaved caspase-9 (ab2324), total-caspase-9 (ab32539), cleaved PARP (ab32064), total-PARP (P7605), E-cadherin (ab1416), N-cadherin (ab76057), MMP2 (ab97779), MMP7 (ab205525), OCT4 (ab181557), Nanog (ab218524), Twist (ab187008), GLI2 (ab26056), GLI1 (ab240088), GLI3 (ab6050), GLI4 (ab158532), and GAPDH (ab8245), were purchased from Abcam (Cambridge, USA) or Sigma-Aldrich.

### Transwell assay

To assessing invasion and migration of cells, transwell assay was conducted as described^29^.

### Subcellular fractionation

Using NE-PER Nuclear and Cytoplasmic Extraction Reagents (Thermo Fisher Scientific), cytoplasm and nucleus were extracted from HCCLM3 or MHCC-97H cells prior to RT-qPCR.

### Fluorescence in situ hybridization (FISH) analysis

A PARIS kit (Life Technologies) was utilized to separate nuclear and cytosolic fraction. RNA FISH probes were then produced by Bogu based on the manufacturer’s instructions. The assay was performed as previously described^30^.

### RNA immunoprecipitation (RIP) assay

The relationship among circZNF609, miR-15a-5p, miR-15b-5p, and GLI2 was investigated via RIP assay. The experiment process was carried out in strict accordance with instructions^31^.

### RNA pull-down assay

Briefly, miR-15a-5p biotin probe, miR-15a-5p no-biotin probe or miR-15b-5p biotin probe, miR-15b-5p no-biotin probe were separately synthesized by Thermo Fisher Scientific. The biotinylated microRNA (miRNA) was incubated overnight with cell lysates (Millipore, Billerica, MA, USA), followed by adding streptavidin magnetic beads (Millipore). Finally, RT-qPCR was used to detect the expression levels.

### Dual-luciferase reporter assay

The wild-type or mutant binding site of miR-15a/b-5p in circZNF609 sequence was synthesized and sub-cloned into pmirGLO dual-luciferase vector (Promega, Madison, WI, USA). Then, circZNF609-WT/Mut vector was co-transfected with transfected plasmids into cells via Lipofectamine2000 (Invitrogen). After 48 h, Dual-luciferase Reporter assay system (Promega) was employed to evaluate the relative luciferase activity.

### Tumor xenograft model

Six Nude mice (male, aged 4–6 weeks) purchased from Shi Laike Company (Shanghi, China) were randomly divided into two groups. According to the guidelines for the Care and Use of Laboratory Animals of Beijing University, animal experiments were strictly conducted as described previously^32^. After injection for 4 weeks, mice were sacrificed. Tumor was surgically removed, followed by further HE, Ki67, PCNA, E-cadherin, N-cadherin, or OCT4 staining. The in vivo experiments were approved by the Ethics Committee of Sichuan Academy of Medical Sciences and Sichuan Provincial People’s Hospital.

### Statistical analysis

Results were presented as mean ± SD and imported into GraphPad Prism 7.0 (GraphPad Software, La Jolla, CA, USA). All experiments were repeated three times. Student’s *t*-test, one-way or two-way analysis of variance was employed for the differences. *P* < 0.05 indicated the difference was statistically significant. Spearman’s correlation analysis found the correlation between the expression of circZNF609, miR-15a-5p, miR-15b-5p, and GLI2.

## Results

### CircZNF609 is highly expressed and featured with loop structure in HCC

**Fig. 1:**
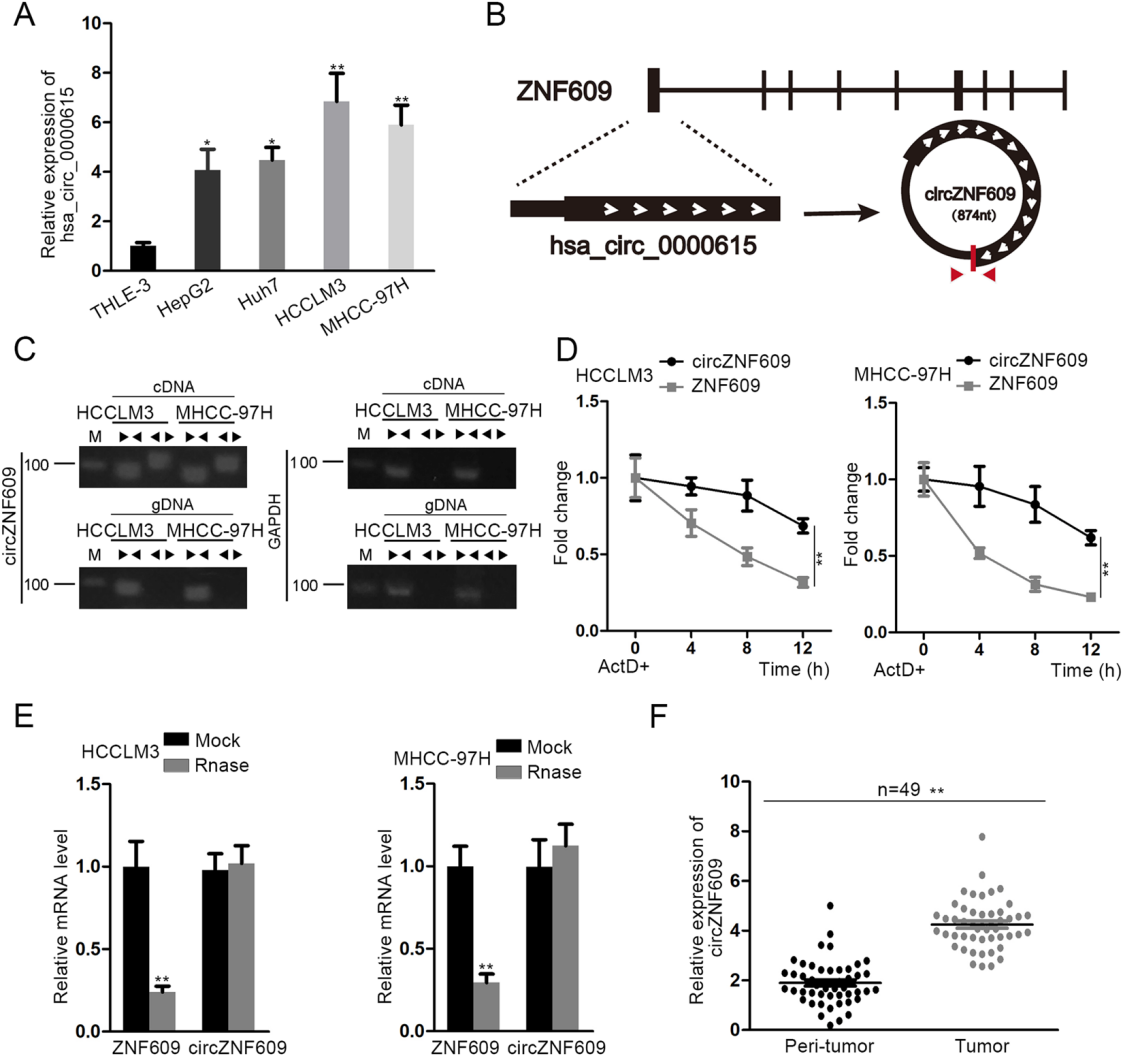
CircZNF609 is highly expressed and featured with loop structure in HCC. **a** RT-qPCR assay was conducted to detect the expression level of circZNF609 in HCC cell lines (HepG2, Huh-7, HCCLM3, and MHCC-97H) and normal human liver epithelial cell line (THLE-3). **b** The genomic location of circZNF609 together with its splicing pattern was displayed. **c** Nucleic acid electrophoresis analysis revealed that divergent primers amplified circular isoform of ZNF609 with cDNA, but not with genomic DNA (gDNA). GAPDH was used as a negative control. **d** The resistance of circZNF609 and ZNF609 mRNA to ActD was detected by RT-qPCR in HCCLM3 and MHCC-97H cells. **e** RT-qPCR assay was carried out to determine the abundance of circZNF609 and linear ZNF609 mRNA in HCCLM3 and MHCC-97H cells treated with RNase R (normalized to mock treatment). **f** The expression status of circZNF609 in HCC tissues and peri-tumor tissues was detected via RT-qPCR. ^*^P < 0.05, ^**^P < 0.01.

The potential role of circZNF609 in HCC remains to be analyzed, though a former investigation has revealed the oncogenic function of hsa_circ_0000615 (circZNF609) on breast cancer tumorigenesis and progression^24^. To further understand the specific function that circZNF609 exerted on the complicated course of HCC development, it is primarily needed to detect the expression level of circZNF609 in HCC cell lines (HepG2, Huh-7, HCCLM3, and MHCC-97H) and normal human liver epithelial cell line (THLE-3). RT-qPCR analysis exhibited a remarkable upregulation of circZNF609 in HCC cell lines compared with that in normal human liver epithelial cell line (Fig. 1a). Prior to functional experiments related to circZNF609 in HCC, the feature of circZNF609 with loop structure need to be testified. First of all, the genomic location of circZNF609, together with its splicing pattern was displayed in Fig. 1b. Subsequently, data from nucleic acid electrophoresis analysis indicated that divergent primers were able to generate the circular isoform of ZNF609 with cDNA but not with gDNA, whereas convergent primers were capable of amplifying the linear isoform of ZNF609 from both cDNA and gDNA in HCCLM3 and MHCC-97H cells (Fig. 1c). After HCCLM3 and MHCC-97H cells were treated with Actinomycin D (ActD, an inhibitor of transcription), ZNF609 mRNA was digested while circZNF609 was apt to be more stable and resistant (Fig. 1d). In addition, ZNF609 mRNA was degraded whereas circZNF609 demonstrated no obvious change in HCCLM3 and MHCC-97H cells after treatment with RNase R (Fig. 1e). Last but not least, RT-qPCR was utilized to examine the expression status of circZNF609 in 49 pairs HCC tissues, as well as peri-tumor tissues and revealed an evidently increased expression of circZNF609 in HCC tissues in comparison with that in peri-tumor (Fig. 1f). Taken together, circZNF609 is overexpressed and characterized with loop structure in HCC.

### Downregulation of circZNF609 impairs HCC cell proliferation, metastasis, and stemness

**Fig. 2:**
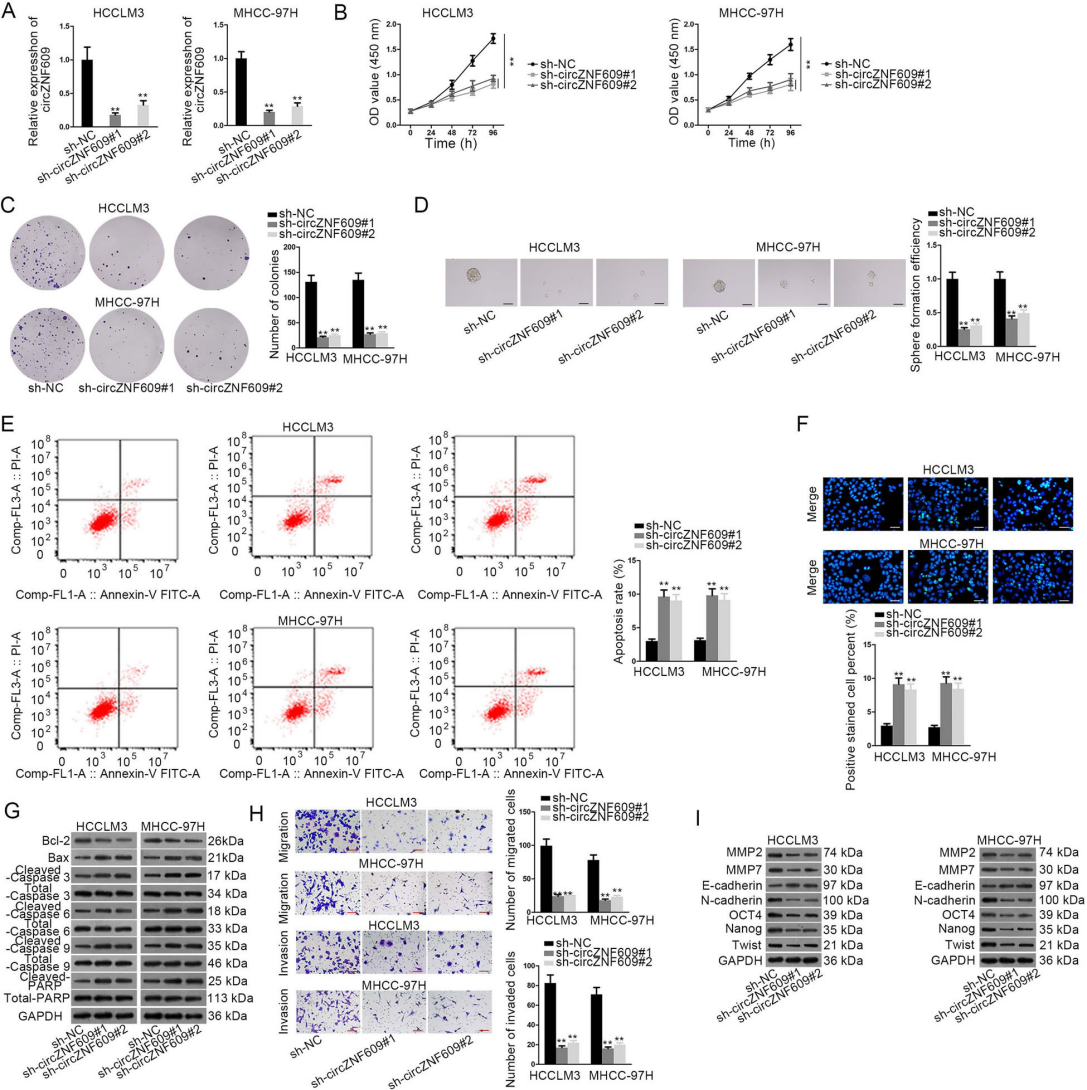
Downregulation of circZNF609 impairs HCC cell proliferation, metastasis, and stemness. **a** The knockdown efficiency of circZNF609 was measured by RT-qPCR in HCCLM3 and MHCC-97H cells. **b**, **c** CCK-8 and colony formation assays were carried out to measure the proliferation ability of transfected cells. **d** Sphere formation assay was used to evaluate cell sphere formation ability in HCCLM3 and MHCC-97H cells transfected with different plasmids. **e**–**g** The apoptosis capability of transfected cells was analyzed by flow cytometry, TUNEL, and western blot analyses. **h** Transwell assay was performed to examine the migration and invasion abilities of transfected cells. I Western blot analysis depicted that knocking down circZNF609 could suppress cell metastasis, EMT, and stemness. ^**^P < 0.01.

After realizing the significant upregulation of circZNF609 expression in HCC tissues and cells and the high stability of circZNF609 due to its circular structure, we decided to further explore the underlying biological role and molecular mechanism of circZNF609 in HCC. Prior to loss-of-function assays, the knockdown efficiency of circZNF609 ought to be measured by RT-qPCR at first in HCCLM3 and MHCC-97H cells. Moreover, the result showed that compared with negative control, circZNF609 expression was observably downregulated in sh-circZNF609#1/2-transfected cells (Fig. 2a). Afterwards, cell proliferation ability was evaluated by CCK-8 and colony formation assays in HCCLM3 and MHCC-97H cells transfected with sh-circZNF609#1/2 or sh-NC. The result uncovered that knockdown of circZNF609 suppressed cell proliferation (Fig. 2b, c). Additionally, the sphere formation ability of HCCLM3 and MHCC-97H cells was restrained by circZNF609 deficiency (Fig. 2d). Besides, the apoptosis capability of transfected cells was evaluated by flow cytometry and TUNEL analyses and confirmed to be enhanced by circZNF609 depletion (Fig. 2e, f). Western blot analysis later depicted that circZNF609 downregulation led to a decreased expression of Bcl-2 but an elevated expression of Bax, cleaved caspase-3, cleaved caspase-6, cleaved caspase-9 and cleaved PARP, indicating a promoting function of circZNF609 knockdown on cell apoptosis (Fig. 2g). The follow-up transwell assay demonstrated that circZNF609 silencing repressed cell migration and invasion in HCCLM3 and MHCC-97H cells (Fig. 2h). Finally, it was obtained from western blot analysis that the expression of MMP2, MMP7, N-cadherin, OCT4, Nanog, and Twist was reduced by silenced circZNF609 while the expression of E-cadherin was on the contrary, suggesting that knocking down circZNF609 could suppress cell metastasis, EMT and stemness (Fig. 2i). In brief, knocking down circZNF609 impairs HCC cell proliferation, metastasis and stemness.

### CircZNF609 activates Hedgehog pathway to regulate HCC progression

**Fig. 3:**
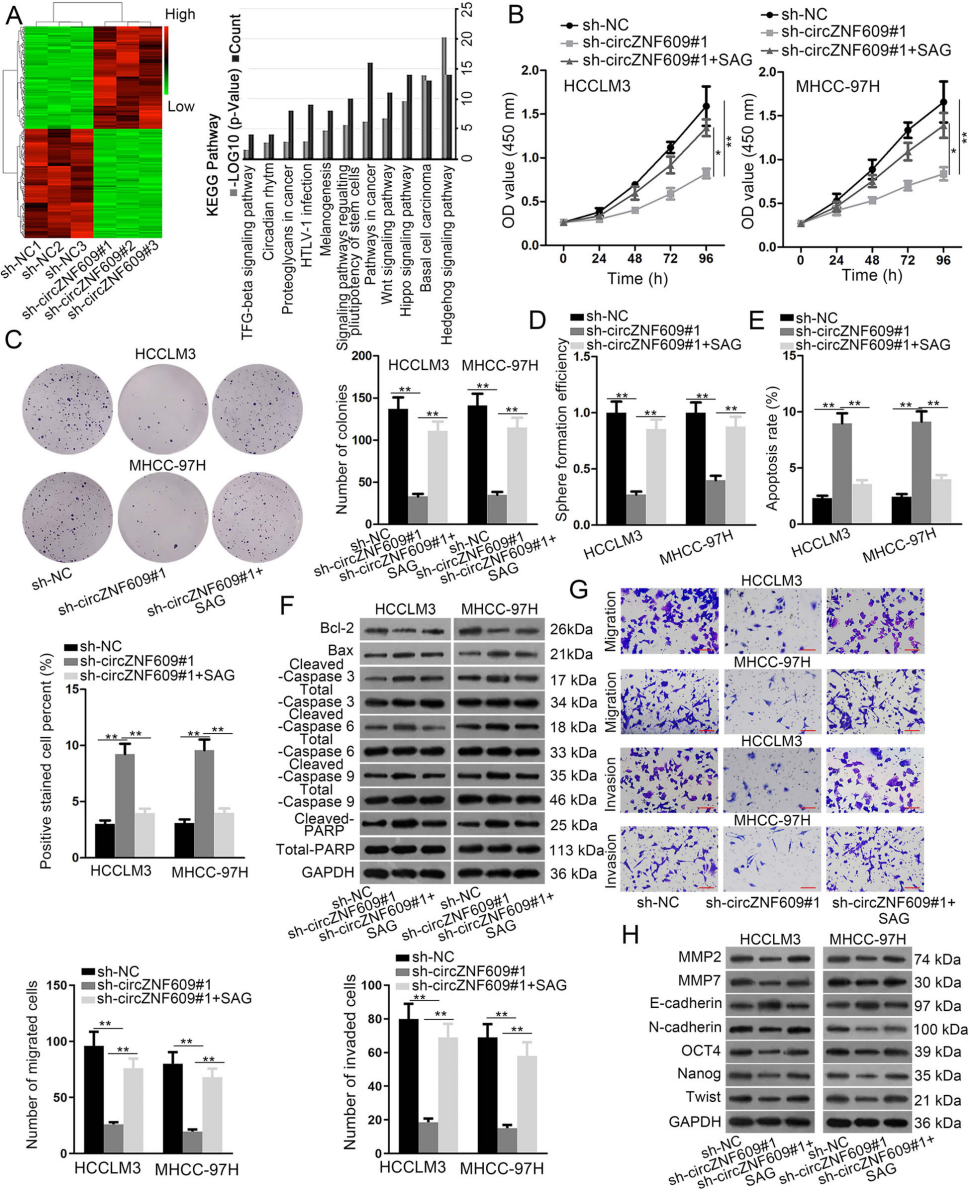
CircZNF609 activates Hedgehog pathway to regulate HCC progression. **a** Microarray and KEGG analyses were applied to detect the expression of the downstream target genes of circZNF609 after circZNF609 was silenced. The result suggested that genes with differential differences in expression were closely related to Hedgehog pathway. **b**, **c** The proliferation ability of HCCLM3 and MHCC-97H cells transfected with different plasmids was measured by CCK-8 and colony formation assays. **d** Cell sphere formation ability in transfected cells was assessed by sphere formation assay. **e**, **f** Flow cytometry, TUNEL, and western blot analyses were employed to analyze the apoptosis capability of transfected cells. **g** Transwell assay was used to examine the migration and invasion capabilities of transfected cells. **h** Cell metastasis, EMT, and stemness in transfected cells were examined by western blot analysis. ^*^P < 0.05, ^**^P < 0.01.

To investigate the possible regulatory mechanism of circZNF609 in HCC, we silenced circZNF609, and then adopted microarray and KEGG analyses to detect the expression of its downstream target genes. The result suggested that genes with differential differences in expression were closely related to Hedgehog pathway (Fig. 3a). Subsequently, SAG (an agonist of Hedgehog pathway) was used to test whether Hedgehog pathway was involved in the regulation of circZNF609 on HCC progression. As illustrated in Fig. 3b, c, cell proliferation ability was weakened by knockdown of circZNF609 and then the effect was restored by addition of SAG in HCCLM3 and MHCC-97H cells. Moreover, an attenuated cell sphere formation ability caused by circZNF609 deficiency was reversed after adding SAG to HCCLM3 and MHCC-97H cells (Fig. 3d). Flow cytometry and TUNEL analyses delineated that the addition of SAG countervailed circZNF609 depletion-mediated promoting effect on cell apoptosis (Fig. 3e). Western blot analysis displayed that the addition of SAG reversed the impact that circZNF609 silencing elicited on the expression of apoptosis-linked proteins, indicating that the addition of SAG offset the function of circZNF609 knockdown on cell apoptosis (Fig. 3f). Moreover, transwell assay demonstrated that the restrained abilities of migration and invasion in HCCLM3 and MHCC-97H cells induced by circZNF609 silencing was then reversed by addition of SAG (Fig. 3g). Final western blot analysis showed that the addition of SAG counteracted circZNF609 knockdown-mediated function on the expression of the metastasis-associated proteins (MMP2 and MMP7), EMT-related proteins (E-cadherin, N-cadherin and Twist), and stemness-linked proteins (OCT4 and Nanog), suggesting that the addition of SAG could partially restore circZNF609 downregulation-mediated suppressive effect on cell metastasis, EMT and stemness (Fig. 3h). All these findings reveal that circZNF609 regulates HCC progression via activation of Hedgehog pathway.

### CircZNF609 activates Hedgehog pathway via the regulation of miR-15a-5p/15b-5p and GLI2 expressions in HCC

**Fig. 4:**
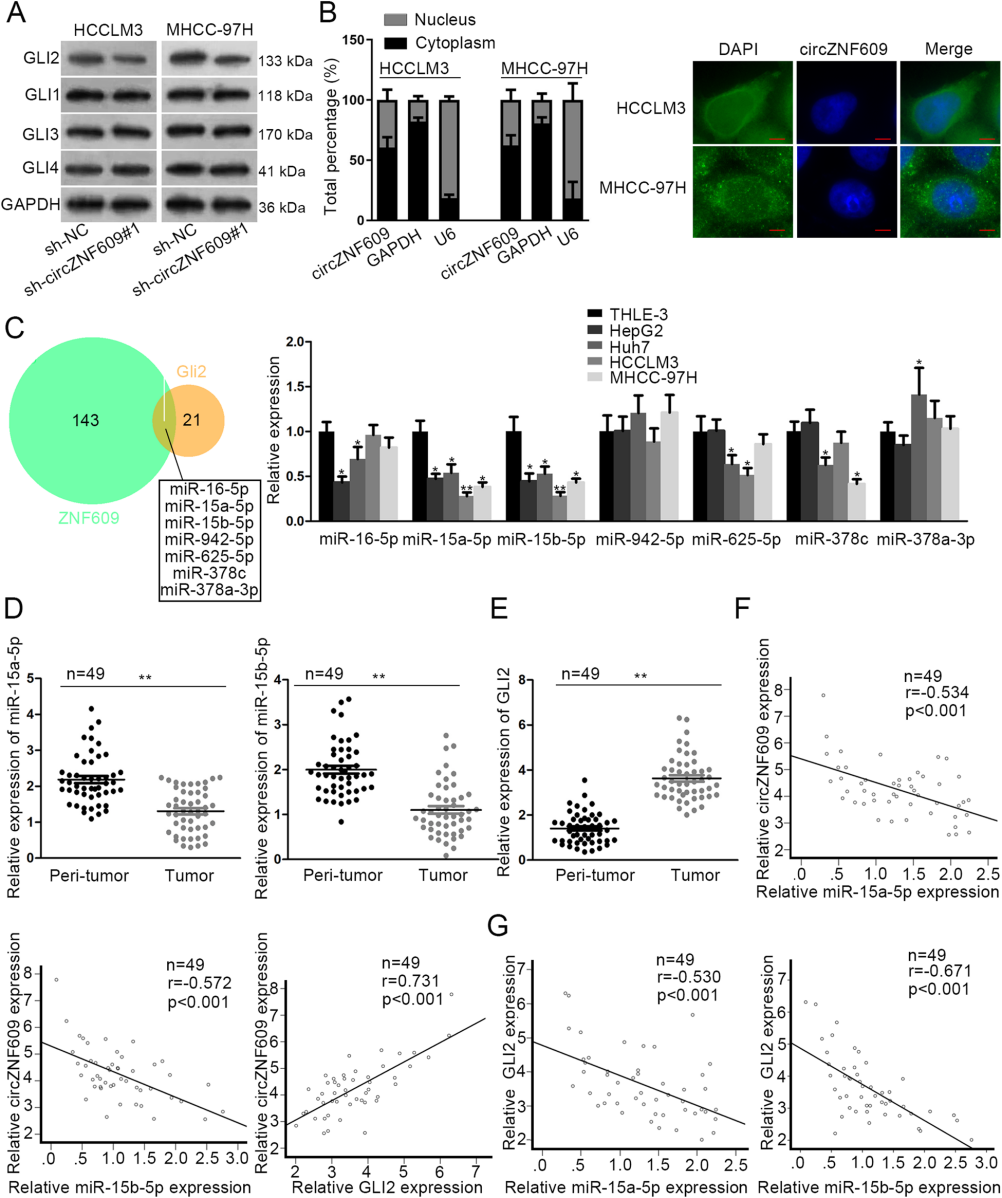
CircZNF609 activates Hedgehog pathway via the regulation of miR-15a-5p/15b-5p and GLI2 expressions in HCC. **a** Western blot analysis was applied to detect the expression of GLI1, GLI2, GLI3, and GLI4 in different groups. **b** Subcellular fractionation and FISH assays were adopted to detect the distribution of circZNF609 in cytoplasm and nucleus. **c** StarBase was utilized and obtained seven miRNAs that not only could bind with circZNF609, but also could bind with GLI2. Moreover, then RT-qPCR was performed to detect the expression of these miRNAs in HCC cell lines and normal human liver epithelial cell line. **d**, **e** The expression of miR-15a-5p/15b-5p and GLI2 was detected in HCC tissues and peri-tumor. **f**, **g** The correlation between miR-15a-5p/15b-5p and circZNF609 (or GLI2), together with the correlation between circZNF609 and GLI2 was analyzed by Spearman’s correlation analysis. ^*^P < 0.05, ^**^P < 0.01.

To further understand how circZNF609 activated Hedgehog pathway in the regulation of HCC development, western blot analysis was conducted at first to detect the expression of several proteins related to Hedgehog pathway and uncovered that only the expression of GLI2 (a key protein molecule concerning Hedgehog pathway) was lowered by circZNF609 knockdown in HCCLM3 and MHCC-97H cells (Fig. 4a). Then subcellular fractionation and FISH assays detected that circZNF609 was mainly located in cytoplasm of HCCLM3 and MHCC-97H cells (Fig. 4b). Hence, an assumption was made that circZNF609 might mediate the activation of Hedgehog pathway by post-transcriptionally regulating GLI2 expression via certain miRNA in HCC. Afterwards, starBase was utilized and obtained 7 miRNAs that not only could bind with circZNF609, but also could bind with GLI2. Moreover, then RT-qPCR detected a significant downregulation of miR-15a-5p and miR-15b-5p in four HCC cell lines (Fig. 4c). Furthermore, in comparison with peri-tumor tissues, a distinctly decreased expression of miR-15a-5p and miR-15b-5p, and an obviously elevated expression of GLI2 in HCC tissues were observed (Fig. 4d, e). Moreover, the negative correlation between miR-15a-5p/15b-5p and circZNF609 (or GLI2), as well as the positive correlation between circZNF609 and GLI2 was obtained through Spearman’s correlation analysis (Fig. 4f, g). Besides, silenced circZNF609 resulted in a significant elevation of miR-15a-5p/15b-5p expression in HCCLM3 and MHCC-97H cells (Supplementary Fig. 1A). In sum, circZNF609 activates Hedgehog pathway via the regulation of miR-15a-5p/15b-5p and GLI2 expressions in HCC.

### CircZNF609 mediates HCC progression through the regulation of miR-15a-5p/15b-5p and GLI2 expressions

**Fig. 5:**
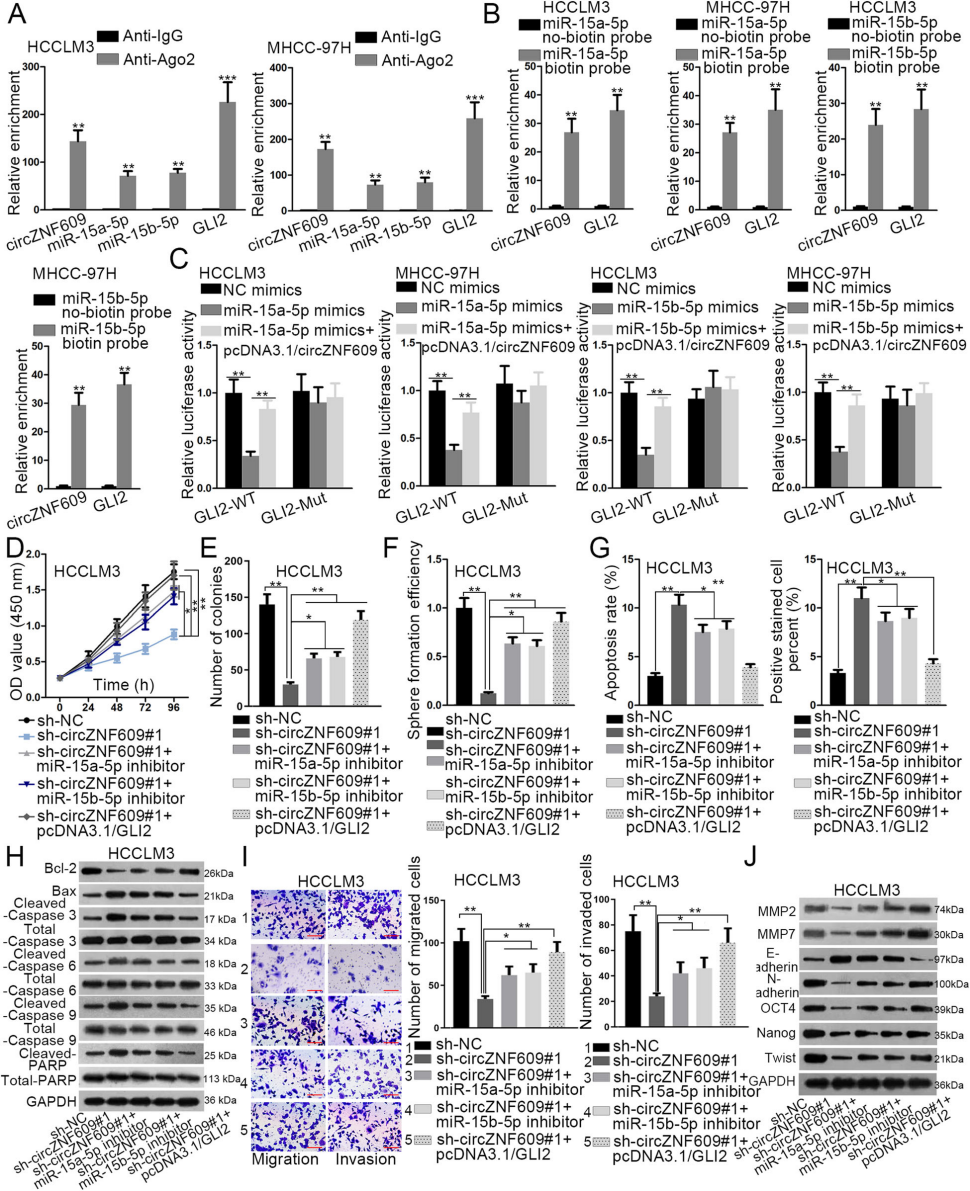
CircZNF609 mediates HCC progression through the regulation of miR-15a-5p/15b-5p and GLI2 expressions. **a**–**c** The relationship among circZNF609, miR-15a-5p/15b-5p, and GLI2 was confirmed by RIP, RNA pull-down, and luciferase reporter assays. **d**, **e** The proliferation ability of transfected cells was measured by CCK-8 and colony formation assays. **f** Cell sphere formation ability in transfected cells was examined by sphere formation assay. **g**, **h** Flow cytometry, TUNEL, and western blot analyses were conducted to evaluate the apoptosis capability of transfected cells. **i** Transwell assay was utilized to examine cell migration and invasion capabilities in transfected cells. **j** Cell metastasis, EMT, and stemness in different groups were examined by western blot analysis. ^*^P < 0.05, ^**^P < 0.01, ^***^P < 0.001.

Proceeding with this study, we planned to further explore the relationship among circZNF609, miR-15a/15b-5p and GLI2 in HCC. First, RIP assay displayed that circZNF609, miR-15a-5p/15b-5p and GLI2 were all enriched in anti-Ago2 group rather than anti-IgG group, suggesting the coexistence of circZNF609, miR-15a/15b-5p and GLI2 in RNA-induced silencing complex (RISC) (Fig. 5a). The following RNA pull-down assay confirmed that miR-15a-5p/15b-5p could bind with circZNF609 (or GLI2) in HCCLM3 and MHCC-97H cells (Fig. 5b). Besides, luciferase activity assay showed that circZNF609 overexpression largely rescued miR-15a-5p/15b-5p mimics-mediated negative effects on the luciferase activity of GLI2-WT reporter, whereas the luciferase activity of GLI2-Mut reporter exhibited no evident change after the overexpression of miR-15a-5p/15b-5p or circZNF609 (Fig. 5c). To further prove the molecular mechanism in HCC, rescue assays were employed in the following part. As displayed in Fig. 5d, e, GLI2 overexpression or miR-15a-5p/15b-5p inhibition rescued circZNF609 depletion-mediated inhibitory function on cell proliferation in different degrees. Similarly, GLI2 upregulation or miR-15a-5p/15b-5p suppression recovered circZNF609 depletion-mediated inhibitive effect on cell sphere formation ability to different degrees (Fig. 5f). Likewise, GLI2 overexpression or miR-15a-5p/15b-5p inhibitor offset circZNF609 depletion-mediated promoting function on cell apoptosis at different level (Fig. 5g). Additionally, overexpression of GLI2 or inhibition of miR-15a-5p/15b-5p countervailed circZNF609 depletion-mediated effect on the expression of apoptosis-linked proteins at different level (Fig. 5h). Furthermore, cell migration and invasion abilities in HCCLM3 cells was repressed by circZNF609 deficiency, which was then restored to varying degrees by GLI2 upregulation or miR-15a-5p/15b-5p suppression (Fig. 5i). At last, GLI2 overexpression or miR-15a-5p/15b-5p inhibition counteracted circZNF609 knockdown-mediated function on the expression of the metastasis-associated proteins, EMT-related proteins and stemness-linked proteins in various degrees (Fig. 5j). To sum up, circZNF609 promotes HCC progression through inhibiting miR-15a-5p/15b-5p expression and elevating GLI2 expression.

### CircZNF609 contributes to the in vivo tumorigenesis of HCC

**Fig. 6:**
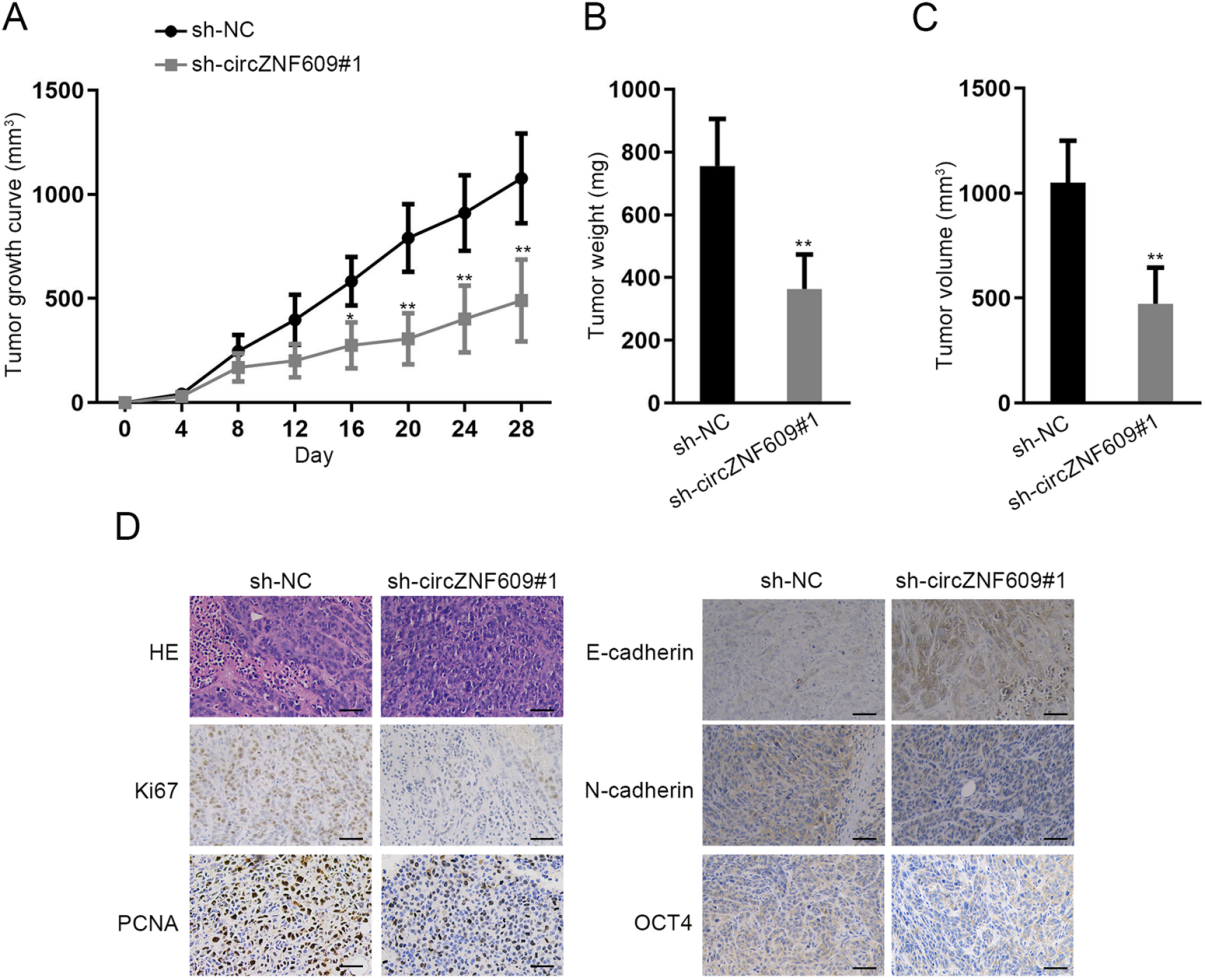
CircZNF609 contributes to the in vivo tumorigenesis of HCC. **a**–**c** HCCLM3 cells transfected with sh-circZNF609#1 or sh-NC were inoculated into the flanks of mice subcutaneously. Then tumor growth, tumor weight, and tumor volume were examined in different groups. **d** IHC assay was used to evaluate the expression of Ki67, PCNA, E-cadherin, N-cadherin, and OCT4 in different groups. ^*^P < 0.05, ^**^P < 0.01.

After conducting assays on the in vitro tumorigenesis of HCC, experiments concerning the in vivo tumorigenesis of HCC were required to further verify the biological role of circZNF609 in HCC. Then HCCLM3 cells transfected with sh-circZNF609#1 or sh-NC were inoculated into the flanks of mice subcutaneously. After injection for 28 days, mice were sacrificed and tumors were removed from mice for further analysis. As illustrated in Fig. 6a, silenced circZNF609 impaired tumor growth. Moreover, circZNF609 depletion decreased tumor weight (Fig. 6b). Besides, a smaller tumor volume induced by downregulation of circZNF609 was observed (Fig. 6c). Further, it was depicted by immunohistochemistry (IHC) assay that circZNF609 knockdown led to a lowered expression of Ki67, PCNA, N-cadherin, OCT4, and an enhanced expression of E-cadherin (Fig. 6d). All these data elucidate that circZNF609 depletion inhibits the in vivo tumorigenesis of HCC.

## Discussion

In the past few years, HCC has been diagnosed as the most prevalent primary hepatic malignant tumor inducing many health problems worldwide^1,2^. Abundant evidence has unveiled the critical impact that circRNA elicits on the initiation and progression of different kinds of diseases, particularly in human malignancies, including breast cancer^18^, osteosarcoma^19^, oral mucosal melanoma^33^, non-small cell lung cancer^20^, and HCC^21^. Although an existing research concerning breast cancer have elucidated the key regulatory role of circZNF609 on cell growth and metastasis^24^, the underlying regulatory function of circZNF609 on the tumorigenesis of HCC still needs exploration. Therefore, this study was the first attempt to make clear the function that circZNF609 exerted on HCC tumorigenesis and development. To achieve this purpose, the dramatically upregulated expression of circZNF609 was detected and then circular structure of it was confirmed in HCC, followed by functional assays concerning the biological role of circZNF609 in tumorigenesis and development of HCC. The results elucidated that knockdown of circZNF609 impaired HCC cell proliferation, metastasis and stemness whereas facilitated cell apoptosis.

Subsequently, the downstream target genes of circZNF609 with significant differences in expression were found to be closely related to Hedgehog pathway through microarray and KEGG analyses. Then we speculated that Hedgehog pathway might participate in the regulation of circZNF609 in HCC due to the existing studies, which have revealed that Hedgehog pathway was associated with the tumorigenesis and development of tumors, such as osteosarcoma^34^ and retinoblastoma^35^. Afterwards, SAG (an agonist of Hedgehog pathway) was used to test the above speculation and then a series of experiments delineated that the addition of SAG could partially rescue circZNF609 knockdown-mediated function on HCC cell proliferation, metastasis, stemness, as well as cell apoptosis, which implied that the activation of Hedgehog pathway could promote HCC progression.

Subsequently, GLI2 (a key protein molecule concerning Hedgehog pathway) was found to be lowered by circZNF609 knockdown in HCCLM3 and MHCC-97H cells. Considering the close relation of GLI2 to Hedgehog pathway and the significant role of GLI2 in gastric cancer^36^, GLI2 was treated as another object of this study. In the follow-up test, circZNF609 was discovered to take a bigger proportion in cytoplasm than nucleus in HCC, indicating its post-transcriptional regulation of target gene expression. Thus, we boldly conjectured that circZNF609 might mediate the activation of Hedgehog pathway through post-transcriptional regulation of certain miRNA to mediate GLI2 expression in HCC. Then miR-15a-5p and miR-15b-5p were screened out because of their evidently downregulation in HCC cells. Following assays elucidated that there was a negative correlation between miR-15a-5p/15b-5p and circZNF609 (or GLI2), and a positive correlation between circZNF609 and GLI2 in HCC. Besides, miR-15a-5p/15b-5p was validated to bind with circZNF609 (or GLI2) in HCC. To further proof the molecular mechanism of circZNF609 in HCC, rescue experiments were applied and revealed that GLI2 overexpression or miR-15a-5p/15b-5p inhibition rescued circZNF609 silencing-mediated function on HCC progression in different degrees. Final in vivo assays further confirmed the oncogenic role of circZNF609 in the in vivo tumorigenesis of HCC.

In conclusion, circZNF609 enhances HCC cell proliferation, metastasis, and stemness by activating the Hedgehog pathway through the regulation of miR-15a-5p/15b-5p and GLI2 expressions, providing a new clue for investigators to explore better therapies for HCC.

## Supplementary information

Supplementary Figure legends

Supplementary Figure 1
