# Supplementary material for: CircZNF609 enhances hepatocellular carcinoma cell proliferation, metastasis, and stemness by activating the Hedgehog pathway through the regulation of miR-15a-5p/15b-5p and GLI2 expressions
Source: Cell Death Dis. 2020 May 12;11(5):358. doi: 10.1038/s41419-020-2441-0 (PMC7217914; doi:10.1038/s41419-020-2441-0)
Supplement: Supplementary file 1 — Supplementary Figure legends [file 41419_2020_2441_MOESM1_ESM.docx]

**Supplementary Figure 1**

(A) The expression of miR-15a-5p/15b-5p was detected via RT-qPCR in HCCLM3 and MHCC-97H cells transfected with sh-circZNF609#1 or sh-NC. ^**^P < 0.01.
