# Supplementary figures and images for: CircZNF609 enhances hepatocellular carcinoma cell proliferation, metastasis, and stemness by activating the Hedgehog pathway through the regulation of miR-15a-5p/15b-5p and GLI2 expressions
Source: Cell Death Dis. 2020 May 12;11(5):358. doi: 10.1038/s41419-020-2441-0 (PMC7217914; doi:10.1038/s41419-020-2441-0)

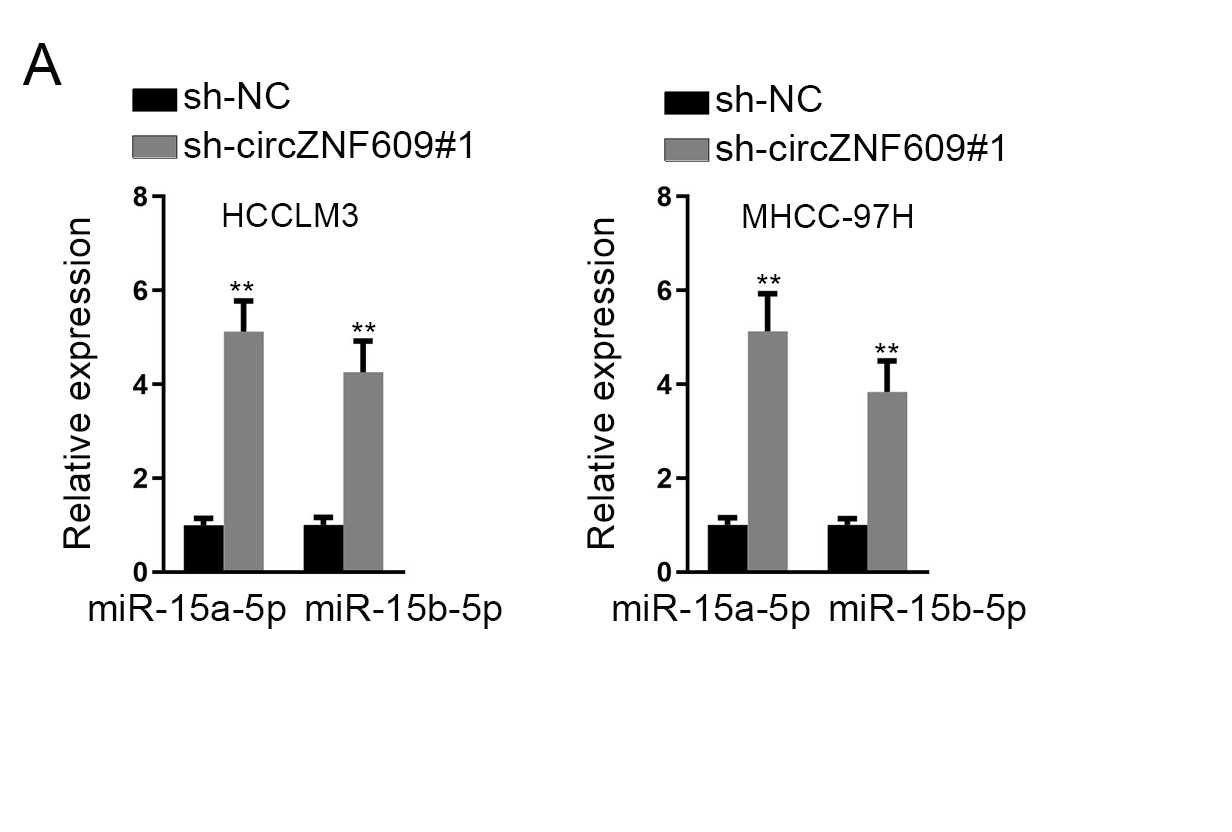

Supplement: Supplementary file 2 — Supplementary Figure 1 [file 41419_2020_2441_MOESM2_ESM.tif]
